# Instruction in Psycho Therapy

**Published:** 1914-02-07

**Authors:** Sidney Wilkinson

**Affiliations:** 49 Rodney Street, Liverpool


*To the Editor of* THE HOSPITAL.

### Instruction in Psycho-Therapy.

*To the Editor of* THE HOSPITAL.

SIR,—It must be apparent to every practitioner of medicine that there is an increasing number of cases, of a purely functional nature, that remain untouched by physical methods of treatment and that drift helplessly from one doctor to another, and from hospital to hospital, seeking relief from troubles which, though they may have a basis in the imagination, are nevertheless of paramount importance to the patient, and are productive of suffering which is no less, and is frequently more, than that which is the outcome of organic disease.

Even organic diseases, which are not progressive, except on the mental side, can frequently be alleviated, and the distress arrested, by psycho-therapy. In fact, many organic diseases of long duration are rendered more difficult to bear by the superimposing of psychical symptoms.

Many of these cases occur in the well-to-do classes, and may be regarded as the penalty of luxury, excesses, and nervous strain, and, when such is the case, they have plenty of means of obtaining treatment by visits to other towns, if necessary. But the poor are by no means without their worries and irregularities, and when this is the case they are in a worse state than with any other class of complaint known.

In the treatment of all other cases the poor have equal facilities with the rich and far better than the middle classes.

Only the very poor, and the rich, can usually obtain the luxuries of radium, $x$-rays, serum treatment, light treatment, electrical treatment, and all the exact analyses and precise diagnostic aids which an up-to-date hospital can offer.

Until fourteen months ago, however, there was no institution in the United Kingdom where treatment by mental suggestion could be obtained as a routine thing, and unfortunate sufferers in thousands have drifted into the hands of quacks, and fortunes have been made out of the shillings of the poor sufferers from nervous troubles.

Without an institution where the poor can be received for treatment, in a more or less collective manner, there can be no provision for these unfortunates, for, however desirous the physician may be to help, the demand upon his time is too great, psycho-therapeutic treatment being a more lengthy process than prescribing.

Instructions in normal psychology are given in our Universities, yet in no hospital with which I am acquainted in this country is any training given in psycho-therapy, and when seen the methods are regarded with the curiosity of a novelty. The result is that the young and up-to-date practitioner must gather his information from books, or visit one of the numerous clinics on the Continent. That there is need for enlightenment is obvious, there being ample scope in private practice for the use of suggestive treatment.

Psycho-analysis is too lengthy and complex for anyone but the specialist, but simple suggestion can be rapidly used in the general round of visits, frequently with marked effect.

The old *placebo* is but the clumsy employment of suggestion, when it could be more rationally and effectively used with the patients' co-operation in one of the ways scientifically recognised. Instruction on these subjects is what we so much need. I have often heard the remark at a bedside clinic, "This is a case for the psycho-therapist's help," and the case is then proceeded with on the usual drug and dietetic lines, and the student is left to find out what these methods are for himself. In Liverpool we have made a move in this direction, and, though at present we do not give systematic lectures, medical men and students are welcomed to watch the work and to glean all the information we can give them, whilst an average of fifty treatments weekly are given and much good is accomplished. Dr. Edwin Ash's nerve clinic in London is the only one with which I am acquainted where treatment and lectures are regularly combined.

Since Liverpool started the ball rolling London has followed with its psychological clinic, which received a hearty send off, with many eminent psychologists to back it, whilst other centres are making inquiries with a similar view.

May success attend them all. Yours faithfully,

Sidney Wilkinson.

49 Rodney Street,
Liverpool, February 2, 1914.

